# A Unique Three-Tendinous Head Reverse Palmaris Longus: A Case Report

**DOI:** 10.7759/cureus.37735

**Published:** 2023-04-17

**Authors:** Adegbenro O Fakoya, Roshan Mategaonkar, Colton Sellars, Nnenna Mbara

**Affiliations:** 1 Cellular Biology and Anatomy, Louisiana State University Health Sciences Center, Shreveport, USA; 2 Anatomy, University of Medicine and Health Science, Basseterre, KNA; 3 Anatomy, University of Medicine and Health Sciences, Basseterre, KNA

**Keywords:** tendinous head, anomaly, reverse palmaris longus, carpal tunnel syndrome, palmaris longus

## Abstract

The Palmaris longus (PL) is a fusiform muscle that is part of the superficial flexors on the anterior compartment of the forearm. It originates from the common flexor tendon at the medial epicondyle of the humerus and inserts at the flexor retinaculum. The Palmaris longus has been reported to present in multiple variations. Some of these variations include agenesis, reversal, and multiple bellies of the muscle. The Palmaris longus is clinically significant as a landmark for carpal tunnel syndrome steroid injection, hand anesthesia, and used as a surgical graft. Medical students at the University of Medicine and Health Sciences, St. Kitts and Nevis, encountered a unique variation of the PL during cadaver dissection. This article explores the exclusiveness of a three-tendinous head reverse PL and what makes it unique compared to similar findings in other reports.

## Introduction

The Palmaris longus (PL) muscle originates from the common flexor tendon on the medial epicondyle and inserts at the flexor retinaculum. This muscle has been documented to present many variations. Around 20 percent of the population will present with an anomaly of the PL. The most common is muscle agenesis, but it can vary widely depending on ethnicity [1]. One of the rarer anomalies and the variation of interest is an inverted or reversed Palmaris longus (RPL), which has its muscle belly closer to the point of insertion as opposed to the typical PL belly, which is close to the muscle origin. The first documented case of RPL was in 1916 [2]. An RPL is when the muscle belly attaches on the volar side of the head over the flexor retinaculum. In this case review, a tri-tendinous RPL was discovered. Most cases of RPL are found by exploratory imaging for hand pain or during cadaver dissection [3]. The findings of RPL are becoming much more common among individuals that use their hands extensively because of the presenting symptoms of carpal tunnel and Guyon's syndrome, where the muscle belly hypertrophy compresses the median and ulnar nerves, respectively [4]. Individuals with RPL can also manifest with a compressed ulnar artery [5]. In general, it is observed that the width and insertion of the RPL provide the etiology of individuals with presenting symptoms [3]. Understanding the variation in the PL is imperative because of its extensive use in surgical grafts. Since the palmaris longus is a weak flexor, it can be used in transplants or resectioning [3]. Individuals with pain presentations may also electively require the surgical removal of the PL [3].

## Case presentation

**Figure 1:**
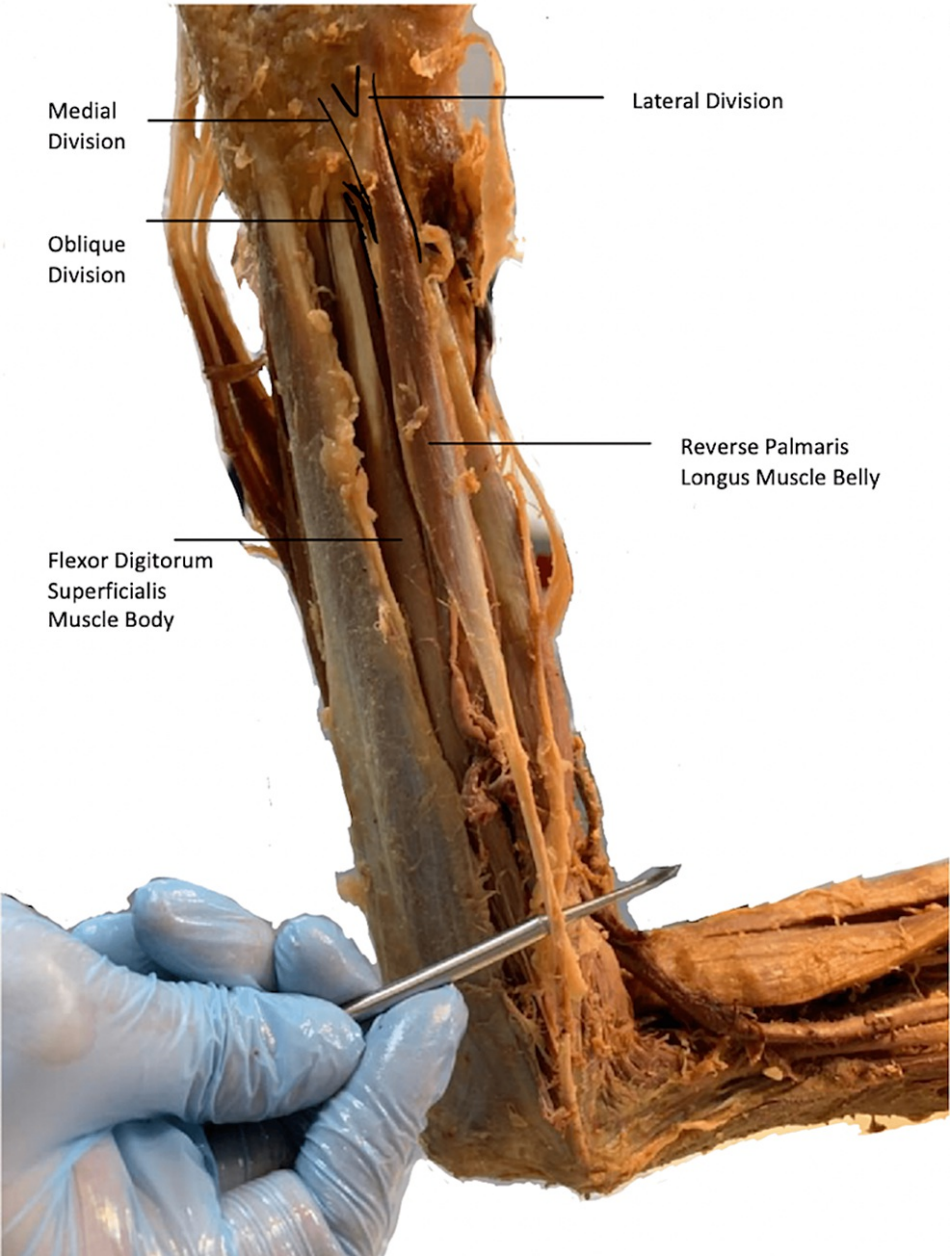
Forearm dissection Three-tendinous head reversed palmaris longus showing lateral, medial, and oblique divisions.

Upon a routine proper anterior forearm dissection of a 68-year-old Caucasian female at the University of Medicine and Health Sciences Anatomy Lab, an anomaly was found in the right PL muscle. During the dissection, we first removed the superficial fascia. After this, we incised and separated the cubital fascia from the antebrachial fascia. With the aid of a probe, the antebrachial fossa was separated from the inferior muscles and detached from its attachments on the radius and ulna. With blunt dissection, we separated the tendons of the superficial layer of the flexor muscles of the right forearm. The muscle bellies of pronator teres (PT), flexor carpi radialis (FCR), flexor carpi ulnaris (FCU), and PL were observed. The tendon of the PL originated from the medial epicondyle, and its three muscle bellies were clearly identified with the aid of a probe. In our dissection, there was a unique pattern where the oblique division of the muscle belly traveled inferiorly with the flexor digitorum superficialis tendon, inferior to the flexor retinaculum, into the carpal tunnel. The medial division, upon observation, was attached to the flexor retinaculum. The lateral division was attached and continuous with the palmar aponeurosis (Figure 1). The PL of the left forearm was normal.

## Discussion

The PL is a fusiform muscle part of the superficial flexors on the anterior compartment of the forearm [5]. It usually originates from the medial epicondyle of the humerus and inserts into the distal half of the flexor retinaculum [6]. The Homeobox (HOX) gene is responsible for developing the accessory PL muscle [7, 8]. The transcription factor Pax3 gene is responsible for the embryological lineage; also, the myogenic transcription factors contain a standard DNA helix linked for binding transcription factors [9]; the transcription factor, Myogenic Regulatory Factors (MRF), specifically MRF4 and myogenin, forms muscle cells [8]. There has been literature that has covered the embryological derivative of agenesis of the muscle; however, the development of the triple head tendon of the RPL can be further explored in the future. According to Riemann et al., a variation can occur where the PL is fused with the flexor digitorum sublimis (Flexor digitorum superficialis) [9].

The PL muscle is relevant for the positioning of the injection of the Carpal Tunnel Steroid Injection (CTSI). CTSI is inserted medially into the Palmaris tendon [10]. However, in the case of the three-headed reverse palmaris, it can cause injury to the PL's muscle belly or tendon heads. Additionally, it could halt the procedure if the muscle belly is too large. Also, the location of the median nerve is deep to the PL tendon in the case of a Hand Anesthesia Procedure of the median nerve (HAP). One of the techniques of HAP is inserting the needle into the ulnar side of the PL tendon [11]. In the case of the three-tendinous head RPL, the needle insertion can pierce the PL muscle belly or one of its tendinous heads. Modifying these procedures in the future due to these anomalies can help improve healthcare delivery and reduce the likelihood of physician error.

The PL tendon lying superficial to the median nerve has been shown to have a clinical impact on the nerve where it might compress it and mimic carpel tunnel syndrome. The median nerve compression has been shown in some cases leading to the longitudinal excision of the muscle. As reported in the case of Patient 1 in Twoon et al., the excision had no wound healing problems postoperatively with full recovery of the patient's functionality [7]. This RPL anomaly can lead to median nerve compression, thus making it imperative for the surgeon to locate the anomaly [12, 8]. Such a scenario has been reported in patients with chronic exertional compartment syndrome, which entails forearm pain due to exertion [12].

 In 1946, the PL did not deem an influential flexor of the wrist [13]. Hence, it is generally used as the tendon transfer that entails the incision of the PL tendon and its connection with the extensor pollicis longus tendon [11]. However, in our case of the three-headed RPL, the three tendons would allow for one of them to be used. Possibly the lateral division attached to the palmar aponeurosis. More reports in the future and modifications of procedures can be developed for the three-headed RPL muscle. The patient's race was mentioned in our study but is absent in other reports. Correlating the race and the incidence of the three-headed RPL may provide insight into the occurrence of the anomaly. 

There have been many cases to date that have reports of the RPL. However, there have been a total of 3 other cases recorded of three-headed RPL muscle. The first mention in literature was the case of a 36-year-old female with a three-headed RPL on the right forearm [5]. Also, the presence or absence of the PL on the opposite forearm was not mentioned (Table 1) [5]. Other variations were not mentioned either. Another case involved a 73-year-old female with an RPL on her left forearm [14]. However, this case report indicated the presence of a PL on the opposite forearm without any variations present (Table 1). Another article reported a unique case of a 21-year-old male with hypertrophied three-headed RPL that caused clinical symptoms (Table 1) [15]. Also, no information regarding the other forearm was mentioned [15]. The lack of classical signs for carpal tunnel syndrome in the 21- and 36-year-olds indicates that there could be another etiology for the pain experienced by the patients (possible ulnar nerve compression) (Table 1) [15].

**Table 1:** Reported cases of three-headed RPL A/-: Absent, At: Atrophy F: Female, Hyp: Hypertrophy, L: Left, M: Male, N: Normal, OF: Opposite forearm, PL: Palmaris, +/P: Present, Longus, R: Right UK: Unknown, V: Variation

Article Name	Sex	R/L forearm	PL OF P/A		V. OF P/A	Age	At/Hyp	Swelling of distal forearm	Tinel’s Sign	Phallen’s Sign	Median N Compressed
Three-headed reversed palmaris longus muscle: a case report and review of the literature, Yildiz et al., 2002 [5].	F	R	UK		UK	36	UK	P	UK	UK	-
Three-headed reversed palmaris longus muscle and its clinical significance, Natsis et al., 2007 [14].	M	R	UK	UK		21	At-/Hyp-	UK	UK	UK	UK
Effort-related compression of median and ulnar nerves as a result of reversed three-headed and hypertrophied palmaris longus muscle with an extension of Guyon's canal, Acikel et al., 2007 [15].	M	R	UK	UK		21	Hyp+	P	P	N	UK

## Conclusions

The reversed palmaris longus has been linked to the incidence of carpal tunnel syndrome since the belly could be localized in the carpal tunnel. Knowledge of this variation is essential to prevent unnecessary carpal tunnel release surgery, in which the median nerve compression may remain unresolved due to the presence of this palmaris longus variant. The triple-tendinous head reversed palmaris longus could facilitate the development of new clinical techniques for Carpal Tunnel Steroid Injection (CTSI) and hand anesthesia. In addition, embryological research on transcription factors like Pex and the MRF family protein can drive future research to find the exact mechanism for the muscle variation for the triple-tendinous reversed palmaris longus.
